# Short-Term Exposure to Urban Air Pollution and Influences on Placental Vascularization Indexes

**DOI:** 10.1289/EHP300

**Published:** 2016-07-06

**Authors:** Karen Hettfleisch, Lisandra Stein Bernardes, Mariana Azevedo Carvalho, Luciana Duzolina Manfré Pastro, Sandra Elisabete Vieira, Silvia R. D. M. Saldiva, Paulo Saldiva, Rossana Pulcineli Vieira Francisco

**Affiliations:** 1 Department of Obstetrics and Gynecology, and; 2 Department of Pediatrics, São Paulo University Medical School, São Paulo, Brazil; 3 Health Institute, State Health Secretariat, São Paulo, Brazil; 4 Institute of Advanced Studies of the University of São Paulo, São Paulo, Brazil

## Abstract

**Background:**

It has been widely demonstrated that air pollution can affect human health and that certain pollutant gases lead to adverse obstetric outcomes, such as preeclampsia and fetal growth restriction.

**Objectives:**

We evaluated the influence of individual maternal exposure to air pollution on placental volume and vascularization evaluated in the first trimester of pregnancy.

**Methods:**

This was a cross-sectional study on low-risk pregnant women living in São Paulo, Brazil. The women carried passive personal NO_2_ and O_3_ monitors in the week preceding evaluation. We employed the virtual organ computer-aided analysis (VOCAL) technique using three-dimensional power Doppler ultrasound to evaluate placental volume and placental vascular indexes [vascularization index (VI), flow index (FI), and vascularization flow index (VFI)]. We analyzed the influence of pollutant levels on log-transformed placental vascularization and volume using multiple regression models.

**Results:**

We evaluated 229 patients. Increased NO_2_ levels had a significant negative association with log of VI (*p* = 0.020 and beta = –0.153) and VFI (*p* = 0.024 and beta = –0.151). NO_2_ and O_3_ had no influence on the log of placental volume or FI.

**Conclusions:**

NO_2_, an estimator of primary air pollutants, was significantly associated with diminished VI and VFI in the first trimester of pregnancy.

**Citation:**

Hettfleisch K, Bernardes LS, Carvalho MA, Pastro LD, Vieira SE, Saldiva SR, Saldiva P, Francisco RP. 2017. Short-term exposure to urban air pollution and influences on placental vascularization indexes. Environ Health Perspect 125:753–759; http://dx.doi.org/10.1289/EHP300

## Introduction

Some air pollutants such as carbon monoxide, nitrogen dioxide (NO_2_), sulfur dioxide, ozone (O_3_), lead, hydrocarbons, and particulate matter are capable of affecting human health (Brook et al. 2010; Costa et al. 2014; de Oliveira et al. 2014; Mayhew 2009; Mirowsky and Gordon 2015; Olmo et al. 2011). Certain pollutant gases lead to adverse obstetric outcomes such as preeclampsia and fetal growth restriction (Olsson et al. 2013; Pedersen et al. 2014; Romão et al. 2013; Sapkota et al. 2012; Shah et al. 2011; van den Hooven et al. 2011; Yorifuji et al. 2015).

These maternal and fetal complications are consequences of the effects of air pollution on placental function and growth. Indeed, the placenta is an extremely important organ that is responsible for the exchange of gas and fetal nutrients during pregnancy. Maternal air pollution exposure may affect pregnancy by inducing oxidative stress and systemic inflammation, decreasing the degree of placental global DNA methylation, and eliciting suboptimal placentation or placental inflammation (Brook et al. 2010; de Melo et al. 2015; Janssen et al. 2013; Kannan et al. 2006; van den Hooven et al. 2012; Veras et al. 2008). Thereby, the above-described pregnancy complications are suggested to have their origin in abnormal early placentation, which is characterized by impaired trophoblast invasion and a lack of modification of the spiral arteries (Campbell 2007; Kaufmann et al. 2003).

In recent years, a combined method involving three-dimensional (3D) imaging associated with power Doppler ultrasonography was employed to evaluate placental volume and vascularization (Araujo Júnior et al. 2011; Campbell 2007; de Paula et al. 2009; Hafner et al. 2010; Odibo et al. 2011; Pairleitner et al. 1999; Pomorski et al. 2014; Zalud and Shaha 2008). Using 3D power Doppler (3DPD), it is possible to estimate placental volume, flow, and vascularization, and this estimation is related to the real volume, flow, and vascularization observed in the organ evaluated (Morel et al. 2010; Pairleitner et al. 1999; Raine-Fenning et al. 2008a).

Because air pollution affects placentation (Kannan et al. 2006; van den Hooven et al. 2012; Veras et al. 2008), we hypothesized that higher individual exposure to air pollution would lead to diminished placental volume, flow, and vascularization estimated by 3DPD evaluation.

Thus, the aim of our study was to evaluate the influence of individual short-term exposure to urban air pollution on placental volume and vascularization during the first trimester of pregnancy in low-risk patients to investigate one of the potential pathways by which maternal air pollution exposure may cause adverse pregnancy outcomes.

## Methods

### Design

This was a cross-sectional study that was part of a larger cohort [“Impact of exposure to air pollution during intrauterine life and postnatal life on respiratory health of children” (PROCRIAR)] designed to verify the effects of air pollution on the health of the maternal–fetal dyad and children.

We recruited low-risk pregnant women from the Health District of Butantan School in São Paulo from October 2011 to January 2014.

The inclusion criteria consisted of the following: single pregnancy, no maternal diseases, and gestational age between 11 and 13 weeks and 6 days as confirmed by the measurement of the crown-rump length (CRL) in the first trimester ultrasound. Exclusion criteria were twin pregnancy, fetal malformation detected on ultrasound, and inappropriate use of the filter.

After inclusion, we performed individual air pollution exposure and sonographic evaluation as described below.

The sonographer was blinded to the values of air pollution exposure, and the personnel conducting air pollution evaluation were blinded to the ultrasound measurements.

This project was approved by the Research Ethics Committee of the University of São Paulo under the number 132/10. Women who agreed to participate signed the informed consent document.

### Measurement of Exposure to Air Pollution

Energética brand cellulose filters, with a diameter of 37 mm, were used as previously described by our group (de André et al. 2014; Carneiro et al. 2011; Novaes et al. 2007). We evaluated individual short-term exposure to NO_2_ and O_3_. A community health agent delivered the passive personal monitor to the patient 7–18 days before the ultrasound scan. We advised pregnant women to use them out of the bag throughout the day. During the night, the filter was left in the room where the woman sleeps, beside the bed. After the recording period, the personal monitors were returned to the facility where we performed the ultrasound scan: at the Clinical Fetal Medicine of Department of Obstetrics, the hospital school, University of São Paulo, Brazil. The personal monitors were sent to the Laboratory of Experimental Air Pollution, University of São Paulo, Brazil (LPAE), where they were disassembled and stored until the analysis. We analyzed the pollutants separately according to the respective protocols (de André et al. 2014; Carneiro et al. 2011; Novaes et al. 2007). The NO_2_ filters were extracted within 24 hr and analyzed by spectrophotometry. If they could not be analyzed at the same time, they were frozen for a period of 2 weeks. Previous studies used the same method to measure NO_2_ (Carneiro et al. 2011; Novaes et al. 2007). We could analyze the O_3_ filters at any time after exposure. We calculated the difference between the final and initial reflectance of the filters and determined the values after and before exposure, respectively, and corrected for the number of days the patient carried the filters (de André et al. 2014). Each patient carried two filters of NO_2_ and O_3_ at the same time, and we considered the average for our analysis. Details of analyses were published by the same group of LPAE researchers (de André et al. 2014; Carneiro et al. 2011; Novaes et al. 2007). We selected NO_2_ and O_3_ as markers of exposure to ambient pollution for several reasons. First, the passive devices could be used without disrupting the daily activities of the individuals enrolled in the study. Second, NO_2_ is a pollutant produced by almost every combustion process and correlates well with other primary pollutants, and hence it is a good marker of urban pollution (Conceição et al. 2001; da Silva et al. 2006). O_3_ represents a good estimator of the exposure to pollutants generated by photochemical processes. Thus, NO_2_ and O_3_ were considered proxy estimators of the complex mixture that composes urban pollution in the present study.

### Ultrasound Evaluation

All patients underwent an ultrasound examination performed by the same operator (KH). The maternal characteristics analyzed included body mass index (BMI = weight/height^2^) at enrollment, smoking status (considered positive if any number of cigarettes was smoked), parity, alcohol consumption, age, ethnicity, and education level. We started the examination with patients in the semi-Fowler’s position to avoid postural hypotension (Khatib et al. 2014) and performed two-dimensional (2D) ultrasound using a 2D convex 3.5-MHz transducer. Localization of the placenta and fetal anomaly scan were performed according to standard published techniques, and CRL measurements were used in early pregnancy to evaluate fetal biometry (Hadlock 1990).

We performed the placental assessments using a 3DPD ultrasound using the same machine brand and model (Voluson 730 Expert^TM^; General-Electric, Austria) in all evaluations and according to the method described by de Paula et al. (2009). Because ultrasound parameters influence placental vascular indexes (Martins et al. 2010; Raine-Fenning et al. 2008b), we used the same pre-established 3DPD instrument settings in all cases (angio mode: cent; smooth: 4/5; FRQ: low; quality: 16; density: 6; enhancement: 16; balance: GO150; filter: 2; actual power: 2 dB; pulse repetition frequency: 0.9 kHz) (de Paula et al. 2009). In the case of artifacts during the acquisition of volume due to fetal movement, we repeated the capture process until a good pattern quality could be achieved. The parameters evaluated included the placental volume and placental vascular indexes [vascularization index (VI), flow index (FI), and vascularization flow index (VFI)].

When a 3D acquisition is performed, the primary image acquired is called voxels (which are the smallest volume acquired by the machine), and they construct the image. The 3DPD colors this primary image according to the movement detected, which gives us a sense of the blood flow. The VI measures the number of color voxels within the volume and represents the number of vessels in the 3D image. The FI measures the intensity of the color in the voxel and represents the flow within the evaluated volume. The VFI is a combination of the two previous indexes and represents the association of vascularization and flow within the organ evaluated (Pairleitner et al. 1999).

We started the ultrasound examination by the placental location and identified its long axis, using 2D technique, and then we adjusted the volume box to scan the entire placenta. We performed teal-time scanning using a convex volumetric transducer from 4.0 to 8.0 MHz (Voluson 730 Expert^TM^ apparatus) using low speed and an angle of 85° to ensure that data from the entire placenta were collected.

**Figure 1:**
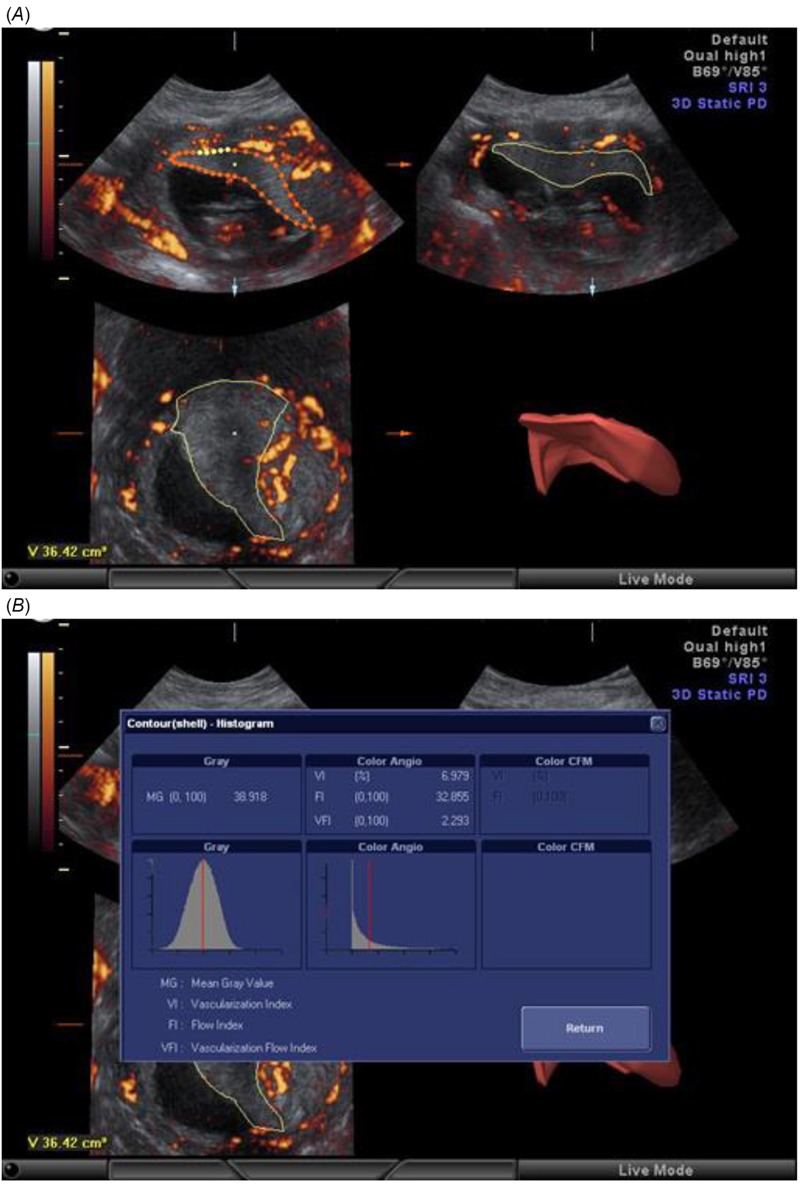
Three-dimensional power Doppler (3DPD) ultrasound. (*A*) Placental capture by 3DPD ultrasonography with the virtual organ computer-aided analysis (VOCAL) method. (*B*) Assessment of placental volume using the rotational technique (VOCAL) and a 3DPD histogram showing the vascular indexes.

After placental acquisition, we calculated the placental volume using virtual organ computer-aided analysis (VOCAL) technique (3D SonoView; GE Medical Systems, Milwaukee, WI, USA) with 30° rotation (Figure 1A) (de Paula et al. 2008). Subsequently, we used 3DPD histogram to obtain the VI, FI, and VFI (Figure 1B) (Pairleitner et al. 1999).

### Statistical Analysis

We used descriptive measures such as mean, median, minimum, maximum, and standard deviation for quantitative variables wherever appropriate. We described the qualitative data using absolute and relative frequencies and percentages. We also evaluated the normal distribution using the Shapiro–Wilk test, histogram graphs, and the observed versus expected values. Because data showed a nonparametric distribution, we used the Spearman’s correlation coefficient to quantify the association between NO_2_ and O_3_ individual exposure.

Logarithmic (log) transformation of the response variables was necessary for normal distribution assumption. To assess the significance of the association between air pollution short-term exposure and the estimate of placental measures (volume, VI, FI, and VFI), we used the linear regression multiple models controlled for BMI, smoking status, parity, localization of the placenta, gestational age, maternal alcohol consumption, maternal age, maternal ethnicity, and maternal education level. We performed a modeling process taking into account the above mentioned control variables. Final models with and without the control variables are presented in order to show the constancy of air pollution influence on the dependent variables (described as Model 1, Model 2, Model 3, and Model 4). We demonstrated the nonstandardized beta-coefficients (95% confidence intervals) and the standardized beta-coefficients (obtained by the standardization of all variables in the model) in all tables to compare the influence of each variable in the regression models (Seber and Lee 2003). Significance was set at *p*-value of < 0.05 or, in the multiple regression models, by the absence of zero in the 95% confidence intervals of beta-coefficients. Since smoking may influence placental vascularization (Jauniaux and Burton 2007; Rizzo et al. 2009), we presented a fourth model evaluating placental vascularization and control variables excluding pregnant smokers. This model was performed to evaluate the influence of air pollution on placental vascularization, regardless of smoking.

For each adjusted model, we performed the residual analysis to verify the linear regression multiple model assumptions. We did not find any evidence of violation of the model assumptions. We also performed the outlier analysis to identify the influential points in the adjusted model. When the influential points were detected, we adjusted the model by removing these points; however, we found no change in the results. We used SPSS for Windows (version 22) to perform the statistical analyses.

## Results

**Figure 2:**
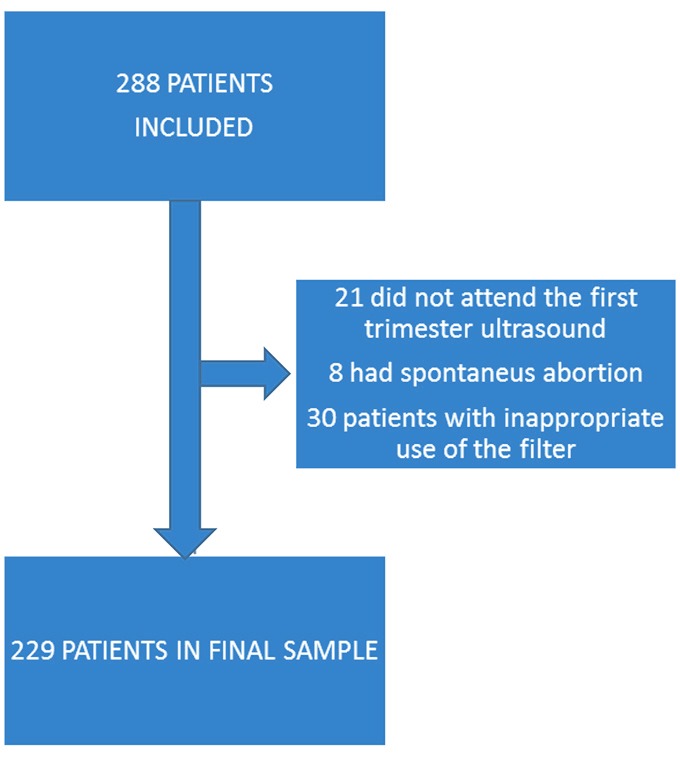
Flow chart of study subjects.

We included a total of 288 patients in this study; 59 (20%) were excluded for the following reasons: 21 women did not attend the first-trimester scan, 8 patients had spontaneous abortions, and 30 patients had inappropriate use of the filter. A total of 229 patients were included in the final sample (Figure 2).

The women carried the filter for a median number of 12 days (range: 7–18 days); 57.6% of the patients had 12 days of exposure and 91.1% of patients stayed with the filter for 10–14 days.

Results of the descriptive analyses of maternal data, placental variables, and pollutants are shown in Table 1. We observed that there was no significant difference in the population included and not included in the study. Table 2 shows that there was no correlation between the pollutants NO_2_ and O_3_.

**Table 1:** Comparison of characteristics of population included in (*n *= 229) and excluded from patients’ groups (*n *= 59). Placental Volumes and Vascularization Indexes, Pollutants (*n *= 229).

Descriptive measures	Included patients	Excluded patients	*p*-Value
Body mass index (kg/m^2^)	26.1 ± 5.6	26.4 ± 5.0	NS (0.478)
Maternal age (years)	25.5 ± 6.2	24.6 ± 6.2	NS (0.350)
Smoking
No	193 (84.3%)	51 (86.4%)	NS (0.696)
Yes	36 (15.7%)	8 (13.6%)
Alcohol consumption
No	206 (90.7%)	52 (88.1%)	NS (0.622)
Yes	21 (9.3%)	7 (11.9%)
Missing	2	0
Maternal ethnicity
White	83 (36.7%)	24 (41.4%)	NS (0.545)
No–white	143 (63.3%)	34 (58.6%)
Missing	3	1
Education level
< 9 years	101 (44.3%)	25 (42.4%)	NS (0.883)
> 9 years	127 (55.7%)	34 (57.3%)
Missing	1	0
Parity
Nulliparous	122 (53.3%)	32 (54.2%)	NS (1.000)
Multiparous	107 (46.7%)	27 (45.8%)
Placental location
No – posterior	125 (54.6%)	—	—
Posterior	104 (45.4%)	—	—
Gestational age (weeks)	12.5 ± 0.64	—	—
Placental volume (cm^3^)	52.2 ± 20.3	—	—
VI (%)	7.7 ± 6.2	—	—
FI	35.7 ± 5.4	—	—
VFI	2.7 ± 2.0	—	—
NO_2_ (μg/m^3^)	40.50 ± 7.72	—	—
O_3_ (μg/m^3^)	8.22 ± 1.15	—	—
Note: Data are mean ± SD or *N* (%). FI, flow index; NO_2_, nitrogen dioxide; NS, not significant; O_3_, ozone; SD, standard deviation; VFI, vascularization flow index; VI, vascularization index. —, data not available.			

**Table 2:** Spearman’s correlation coefficient between the measured pollutants in the first trimester.

	O_3_
NO_2_	0.088
Note: NO_2_, nitrogen dioxide; O_3_, ozone.	

The association between the pollutants NO_2_ and O_3_ and the placenta is shown in Tables 3–6, considering as the outcome variables the placental volume, VI, FI, and VFI, respectively.

**Table 3:** Estimates of the effects of the NO_2_ and O_3_ and significant control variables on placental volume (*n* = 229).

Placental variable/ pollutant and SV	Log volume			
	SB	*p*-Value	SB	95% CI
Model 1^*a*^ (*n* = 228)
NO_2_	0.061	0.315	0.001	–0.001, 0.004
O_3_	0.016	0.790	0.005	–0.034, 0.044
GA	0.454	< 0.001	0.271	0.201, 0.341
Model 2^*b*^ (*n* = 228)
NO_2_	0.070	0.246	0.002	–0.001, 0.004
O_3_	0.018	0.759	0.006	–0.033, 0.045
GA	0.459	< 0.001	0.274	0.204, 0.345
Model 3^*c*^ (*n* = 222)
NO_2_	0.080	0.200	0.002	–0.001, 0.004
O_3_	0.007	0.909	0.002	–0.037, 0.042
GA	0.446	< 0.001	0.264	0.192, 0.336
Model 4^*d*^ (*n* = 187)
NO_2_	0.031	0.658	0.001	–0.002, 0.003
O_3_	0.018	0.793	0.005	–0.035, 0.046
GA	0.433	< 0.001	0.248	0.171, 0.325
Note: CI, confidence intervals; GA, gestational age; Log, logarithm; NO_2_, nitrogen dioxide; O_3_, ozone; SB, standardized beta; SV, significant variable. ^***a***^Model 1: Exposure to both pollutants, controlling for gestational age of fetus. ^***b***^Model 2: Exposure to both pollutants, controlling for gestational age of fetus, body mass index (BMI), parity, smoking, and placental location. ^***c***^Model 3: Exposure to both pollutants, controlling for gestational age of fetus, BMI, parity, smoking, placental location, maternal alcohol consumption, maternal age, maternal ethnicity, and maternal education level. ^***d***^Model 4: Exposure to both pollutants, controlling for gestational age of fetus, BMI, parity, placental location, maternal alcohol consumption, maternal age, maternal ethnicity, and maternal education level.				

Exposure to NO_2_ had a negative association with the log of VI and VFI (Tables 4 and 6). It is important to notice that the fourth model, excluding pregnant smokers, showed similar results to the other models, confirming the consistency of the influence of NO_2_ on these parameters. Similarly, in the models including smokers, there was no association of NO_2_ and O_3_ with the log of placental volume and FI.

**Table 4:** Estimates of the effects of the NO_2_ and O_3_ and significant control variables on placental vascularization index (*n* = 229).

Placental variable/ pollutant and SV	Log VI			
	SB	*p*-Value	SB	95% CI
Model 1^*a*^ (*n* = 228)
NO_2_	–0.168	0.013	–0.008	–0.014, –0.002
O_3_	0.025	0.708	0.017	–0.073, 0.108
Model 2^*b*^ (*n* = 228)
NO_2_	–0.153	0.020	–0.007	–0.013, –0.001
O_3_	0.013	0.842	0.009	–0.079, 0.096
BMI	0.268	< 0.001	0.038	0.020, 0.056
Model 3^*c*^ (*n* = 222)
NO_2_	–0.137	0.042	–0.006	–0.012, –0.0002
O_3_	0.012	0.851	0.009	–0.082, 0.099
BMI	0.280	< 0.001	0.040	0.021, 0.059
Model 4^*d*^ (*n* = 187)
NO_2_	–0.213	0.004	–0.009	–0.015, –0.003
O_3_	0.056	0.430	0.036	–0.054, 0.126
BMI	0.239	0.001	0.034	0.014, 0.055
Note: BMI, body mass index; CI, confidence interval; Log, logarithm; NO_2_, nitrogen dioxide; O_3_, ozone; SB, standardized beta; SV, significant variable; VI, vascularization index. ^***a***^Model 1: Exposure to both pollutants, controlling for gestational age of fetus. ^***b***^Model 2: Exposure to both pollutants, controlling for gestational age of fetus, body mass index (BMI), parity, smoking, and placental location. ^***c***^Model 3: Exposure to both pollutants, controlling for gestational age of fetus, BMI, parity, smoking, placental location, maternal alcohol consumption, maternal age, maternal ethnicity, and maternal education level. ^***d***^Model 4: Exposure to both pollutants, controlling for gestational age of fetus, BMI, parity, placental location, maternal alcohol consumption, maternal age, maternal ethnicity, and maternal education level.				

**Table 5:** Estimates of the effects of the NO_2_ and O_3_ and significant control variables on placental flow index (*n* = 229).

Placental variable/ pollutant and SV	Log FI			
	SB	*p*-Value	SB	95% CI
Model 1^*a*^ (*n* = 228)
NO_2_	–0.001	0.991	–6 × 10^–6^	–0.001, 0.001
O_3_	–0.096	0.154	–0.012	–0.030, 0.005
Model 2^*b*^ (*n* = 228)
NO_2_	0.006	0.927	4.9 × 10^–5^	–0.001, 0.001
O_3_	–0.079	0.197	–0.010	–0.026, 0.005
BMI	–0.430	< 0.001	–0.011	–0.015, –0.008
Parity	0.135	0.037	0.040	0.002, 0.078
Model 3^*c*^ (*n* = 222)
NO_2_	0.011	0.858	9.7 × 10^–5^	–0.001, 0.001
O_3_	–0.073	0.246	–0.009	–0.026, 0.007
BMI	–0.427	< 0.001	–0.011	–0.015, –0.008
Parity	0.190	0.011	0.057	0.013, 0.101
Model 4^*d*^ (*n* = 187)
NO_2_	0.008	0.907	7 × 10^–5^	–0.001, 0.001
O_3_	–0.090	0.185	–0.012	–0.029, 0.006
BMI	–0.432	< 0.001	–0.012	–0.016, –0.009
Parity	0.209	0.007	0.065	0.018, 0.112
Note: BMI, body mass index; CI, confidence intervals; FI, flow index; Log, logarithm; NO_2_, nitrogen dioxide; O_3_, ozone; SB, standardized beta; SV, significant variable. ^***a***^Model 1: Exposure to both pollutants, controlling for gestational age of fetus. ^***b***^Model 2: Exposure to both pollutants, controlling for gestational age of fetus, BMI, parity, smoking, and placental location. ^***c***^Model 3: Exposure to both pollutants, controlling for gestational age of fetus, BMI, parity, smoking, placental location, maternal alcohol consumption, maternal age, maternal ethnicity, and maternal education level. ^***d***^Model 4: Exposure to both pollutants, controlling for gestational age of fetus, BMI, parity, placental location, maternal alcohol consumption, maternal age, maternal ethnicity, and maternal education level.				

**Table 6:** Estimates of the effects of the NO_2_ and O_3_ and significant control variables on placental vascularization and flow index (*n* = 229).

Placental variable/ pollutant and SV	Log VFI			
	SB	*p*-Value	SB	95% CI
Model 1^*a*^ (*n* = 228)
NO_2_	–0.167	0.014	–0.008	–0.014, –0.002
O_3_	0.007	0.913	0.005	–0.086, 0.096
Model 2^*b*^ (*n* = 228)
NO_2_	–0.151	0.024	–0.007	–0.013, –0.001
O_3_	–0.002	0.981	–0.001	–0.091, 0.089
BMI	0.185	0.006	0.026	0.008, 0.045
Model 3^*c*^ (*n* = 222)
NO_2_	–0.136	0.048	–0.006	–0.012, –5.5 × 10^–5^
O_3_	–0.001	0.982	–0.001	–0.094, 0.092
BMI	0.196	0.005	0.028	0.009, 0.048
Model 4^*d*^ (*n* = 187)
NO_2_	–0.211	0.004	–0.009	–0.016, –0.003
O_3_	0.037	0.601	0.025	–0.069, 0.118
Note: BMI, body mass index; CI, confidence intervals; Log, logarithm; NO_2_, nitrogen dioxide; O_3_, ozone; SB, standardized beta; SV, significant variable; VFI, vascularization and flow index. ^***a***^Model 1: Exposure to both pollutants, controlling for gestational age of fetus. ^***b***^Model 2: Exposure to both pollutants, controlling for gestational age of fetus, BMI, parity, smoking, and placental location. ^***c***^Model 3: Exposure to both pollutants, controlling for gestational age of fetus, BMI, parity, smoking, placental location, maternal alcohol consumption, maternal age, maternal ethnicity, and maternal education level. ^***d***^Model 4: Exposure to both pollutants, controlling for gestational age of fetus., BMI, parity, placental location, maternal alcohol consumption, maternal age, maternal ethnicity, and maternal education level.				

Evaluation of the control variables showed that gestational age had a positive association with placental volume. BMI was also associated with all the vascular indexes. Multiparty had a positive association with IF. In the first trimester, placental location and tobacco use showed no influence on the placental volume and vascular indexes. Furthermore, alcohol consumption, maternal age, ethnicity, and education level had no significant association with the outcome variables.

## Discussion

To the best of our knowledge, this is the first study to investigate the effect of air pollution on vascular indexes and placental volume and to consider the individual short-term exposure to pollutants in the first trimester of pregnancy. In the present study, exposure to NO_2_ had a negative influence on VI and VFI. This finding suggests that the placental vasculature can be impaired by exposure to air pollution at ambient levels, specifically to NO_2_, which correlates well with other primary pollutants and is a good marker of urban pollution (Conceição et al. 2001; da Silva et al. 2006). We observed that the influence of NO_2_ was present for VI and VFI, but not for FI. This influence probably occurs because the number of vessels is affected, whereas the flow is not affected in this specific point of gestation. Studies that have evaluated placental vascularization in the first trimester and have predicted pregnancy complications observed that women with diminished VI and VFI showed an increased tendency to have pregnancies that were later complicated with preeclampsia and fetal growth restriction (de Almeida Pimenta et al. 2014; Odeh et al. 2011; Odibo et al. 2011). Short-term exposure to air pollution seems to affect the same indexes, which indicates the possible pathway by which air pollution may provoke these complications (Olsson et al. 2013; Pedersen et al. 2014; Romão et al. 2013; Sapkota et al. 2012; Shah et al. 2011; van den Hooven et al. 2011; Yorifuji et al. 2015).

As already shown in the literature, gestational age had a positive influence on the placental volume (de Paula et al. 2008). On the other hand, we observed that NO_2_ and O_3_ did not have any influence on placental volume at this stage. At the first trimester, vascularization is being formed by branching of immature intermediate villi (Demir et al. 2007; Wang and Zhao 2010), and the major process occurring at this point is vasculogenesis. Placental volume increases more substantially in volume in the second and third trimester (de Paula et al. 2008; Wang and Zhao 2010). This might be why the volume was not influenced in the first trimester by the concentration of NO_2_, but only the placental vascularization.

BMI has an influence on the vascular indexes, as previously demonstrated by other authors (Hafner et al. 2010; Odibo et al. 2011). Similarly, as demonstrated by Zalud and Shaha (2008), multiparity had a positive association with FI. On the other hand, placental location and tobacco use showed no significant effect (de Paula et al. 2009; Guiot et al. 2008; Hafner et al. 2010; Odibo et al. 2011).

Numerous clinical studies conducted around the world have examined the hypothesis that air pollution damages health and, in particular, can negatively affect pregnancy and placental functioning. Possible mechanisms for this association include oxidative stress, inflammation, systemic alterations in the hematocrit and blood viscosity, coagulation, endothelial dysfunction, and hemodynamic responses. These mechanisms are regarded as leading to a loss in placentation and placental dysfunction (Brook et al. 2010; de Melo et al. 2015; Kannan et al. 2006; Slama et al. 2008; Sørensen et al. 2003; Veras et al. 2008). Because adequate placentation and placental functioning are essential for a normal pregnancy, impairment of these processes, reflected by alterations in markers of placental growth and function, represent a risk factor for fetal adverse outcomes (Schlembach et al. 2007; Veras et al. 2008). In their study, van den Hooven et al. (2012) showed that maternal exposure to particulate matter ≤ 10 μm in aerodynamic diameter and NO_2_ exposure were associated with changes in fetal soluble fms-like tyrosine kinase 1 (sFlt-1) and placental growth factor levels at delivery, which is consistent with an anti-angiogenic state. These changes may influence placental development by decreasing vascularization, which may be demonstrated by 3DPD evaluation.

Indeed, these results are consistent with published experimental findings (de Melo et al. 2015; Veras et al. 2008). Veras et al. (2008) showed that pregnant mice exposed to air pollution had decreased placental vasculature, but the fetuses had decreased flow resistance in order to maintain the proper flow. de Melo et al. (2015) demonstrated that pregnant rats exposed to air pollution, before and during pregnancy, had an increase of interleukin-4 in the fetal portion of the placenta, suggesting an anti-inflammatory placental response to previous inflammatory process induced by the pollutants.

The quantification of placental flow and vascularization by 3DPD may vary with ultrasound presets. Therefore, standardization of power Doppler parameters is critical. In our study, all presets were fixed in all evaluations (Huster et al. 2010; Jones et al. 2011; Martins et al. 2010; Raine-Fenning et al. 2008b). Another factor already known to influence the vascular indexes is the distance between the probe and the volume of interest. In our study, we controlled this influence using BMI and placental location, both related to placental depth (Martins et al. 2010). When presets are fixed and the distance between the probe and the structure evaluated is taken into account, this technique has a good reproducibility and has satisfactory correlation with real vascularization of the organ (Bernardes et al. 2011; Huster et al. 2010; Jones et al. 2009, 2011; Raine-Fenning et al. 2003, 2008a).

One limitation of our study is that placental histology was not evaluated to assess the influence of air pollution on actual vascularization. However, because placental indexes correlate with real vascularization and flow (Morel et al. 2010; Pairleitner et al. 1999; Raine-Fenning et al. 2008a), our results suggest that these factors are diminished when the mother is exposed to higher values of NO_2_. Diminished placental vascularization may be the underlying cause of the impaired fetal growth and adverse pregnancy outcomes are related to higher levels of air pollution exposure during pregnancy (Olsson et al. 2013; Pedersen et al. 2013, 2014; Romão et al. 2013; Sapkota et al. 2012; Shah et al. 2011; van den Hooven et al. 2011, 2012; Yorifuji et al. 2015).

Another limitation of this study is the lack of a control group in a non-urban center. However, because exposure to air pollution was measured using individual filters, different lifestyle habits led to different air pollution exposures for each woman evaluated (Steinle et al. 2013).

## Conclusions

This study showed that the placental VI and placental VFI are significantly decreased in the first trimester in pregnant women exposed to higher concentrations of NO_2_, which suggests that this pollutant and other primary pollutants that are associated with NO_2_ influence placentation and decrease placental vascularization. Because placentation permits normal pregnancy and fetal development, these findings suggest that this negative influence may be the underlying cause of pregnancy complications related to short-term air pollution exposure.
